# Potential off-target effects of beta-blockers on gut hormone receptors: *In silico* study including GUT-DOCK—A web service for small-molecule docking

**DOI:** 10.1371/journal.pone.0210705

**Published:** 2019-01-25

**Authors:** Pawel Pasznik, Ewelina Rutkowska, Szymon Niewieczerzal, Judyta Cielecka-Piontek, Dorota Latek

**Affiliations:** 1 Faculty of Chemistry, University of Warsaw, Warsaw, Poland; 2 Department of Pharmacognosy, Faculty of Pharmacy, Poznan University of Medical Sciences, Poznan, Poland; UMR-S1134, INSERM, Université Paris Diderot, INTS, FRANCE

## Abstract

The prolonged use of many currently available drugs results in the severe side effect of the disruption of glucose metabolism leading to type 2 diabetes mellitus (T2DM. Gut hormone receptors including glucagon receptor (GCGR) and the incretin hormone receptors: glucagon-like peptide 1 receptor (GLP1R) and gastric inhibitory polypeptide receptor (GIPR) are important drug targets for the treatment of T2DM, as they play roles in the regulation of glucose and insulin levels and of food intake. In this study, we hypothesized that we could compensate for the negative influences of specific drugs on glucose metabolism by the positive incretin effect enhanced by the off-target interactions with incretin GPCR receptors. As a test case, we chose to examine beta-blockers because beta-adrenergic receptors and incretin receptors are expressed in a similar location, making off-target interactions possible. The binding affinity of drugs for incretin receptors was approximated by using two docking scoring functions of Autodock VINA (GUT-DOCK) and Glide (Schrodinger) and juxtaposing these values with the medical information on drug-induced T2DM. We observed that beta-blockers with the highest theoretical binding affinities for gut hormone receptors were reported as the least harmful to glucose homeostasis in clinical trials. Notably, a recently discovered beta-blocker compound 15 ([4-((2S)-3-(((S)-3-(3-bromophenyl)-1-(methylamino)-1-oxopropan-2-yl)amino)-2-(2-cyclohexyl-2-phenylacetamido)-3-oxopropyl)benzamide was among the top-scoring drugs, potentially supporting its use in the treatment of hypertension in diabetic patients. Our recently developed web service GUT-DOCK (gut-dock.miningmembrane.com) allows for the execution of similar studies for any drug-like molecule. Specifically, users can compute the binding affinities for various class B GPCRs, gut hormone receptors, VIPR1 and PAC1R.

## Introduction

The number of diabetic patients is rapidly increasing, reaching 425 million cases in 2018 [1]. Type 2 diabetes mellitus (T2DM) is the most prevalent form of diabetes. Factors favoring the occurrence of T2DM include obesity, lack of physical activity, disruption of biological rhythm [2] caused, i. a., by iatrogenic factors resulting from pharmacotherapy of chronic diseases [3]. Glucose metabolism can be disturbed by pharmacotherapy on various signaling pathways in three major areas: pancreatic insulin secretion, hepatic glucose production and peripheral tissues insulin sensitivity [3]. It is also well known that specific drug classes, e.g., glucocorticosteroids, thiazides and beta-blockers may induce T2DM more frequently than other drug classes [3, 4]. Nevertheless, the molecular mechanism underlying drug-induced T2DM, including potential off-target interactions [5], is still not fully understood and certainly varies from one drug class to another [3, 6, 7]. Notably, it is crucial to identify the location of the main molecular target (on-target) of a given drug within a cell and/or a tissue in order to trace its off-target interactions associated with the occurrence of side effects [6]. There are many ways to treat drug-induced T2DM, including an optimized polytherapy [8]. Additionally, the broadly understood structure-activity relationship studies can lead to the development of more pharmacologically effective analogs with milder side effects, e.g., beta-1-adrenergic selective blockers vs. non-selective beta-blockers [9]. Additional details on T2DM induced by various drug classes have recently been described in a recent manuscript that is complementary to the current study [4]. This previous study mainly describes T2DM induced by diuretics, steroids and other drugs that were deposited in the SIDER database. The current study is focused only on the beta-blockers drug class.

In both studies, we proposed an *in silico* solution to the drug-induced T2DM problem using the concept of drug repurposing or off-target interactions. Off-target interactions or, in other words, interactions with proteins which are not the intended targets (on-targets) of a specific drug, can be either beneficial (drug repurposing) or unwanted (when they cause adverse drug reactions) [5]. In our study, we hypothesized that the beneficial off-target interactions of a particular drug can compensate for the negative influence of the same drug on other signaling pathways associated with glucose metabolism. As a test case we examined antihypertensive beta-blockers, which are also known to induce new-onset diabetes [9]. We selected gut hormone receptors that are involved in glucose homeostasis regulation as potential off-target proteins [10]. Gut hormone receptors are evolutionarily related to beta-adrenergic receptors (the intended targets of beta-blockers), though they are class B and class A GPCRs, respectively. Recently, the gut hormone receptors GLP1R and GCGR have also been experimentally confirmed as being important for cardiovascular system functioning [11–13].GLP1RIn addition to the gastrointestinal tract, where many drugs are absorbed, gut hormone receptors are also expressed in the membranes of cardiac and vascular cells [14, 15], an expression pattern similar to that of beta-adrenergic receptors. Similar cell and tissue expression patterns of the intended and unintended targets of a drug might favor the occurrence of the off-target interactions [16]. Recently, a relationship has been discovered between GLP-1-based therapies and the abundance of myocardial beta-1 adrenergic receptors, confirming an association between these two signaling pathways [11]. Additionally, studies have evaluated the use of glucagon in treating beta-blocker overdose [13] and heart failure [12]. Gut hormone receptors include: glucagon receptor (GCGR), glucagon-like peptide 1 receptor (GLP1RGLP1R) and gastric inhibitory polypeptide receptor (GIPR). These three GPCRs are also known as glucagon receptors or incretin receptors and are responsible for the regulation of glucose homeostasis and affect insulin secretion [17]. In short, an increase in the concentration of endogenous GIP and GLP-1 peptides positively affects blood insulin levels. Conversely, glucagon increases hepatic glucose production [15]. These effects are commonly described using a single term: the incretin effect. Incretin therapies which affect gut-brain axis signaling, have recently became important second- or third-line T2DM treatment options [15, 17].

In addition to gut hormone receptors we included in this study two other class B GPCRs that which are also partly expressed in the gastrointestinal tract: vasoactive intestinal polypeptide receptor 1 (VIPR1, VPAC1, PACAP-R2) and pituitary adenylate cyclase-activating polypeptide type I receptor (PAC1R, PACAP-R1, ADCYAP1R1PAC1R). The impact of these GPCRs on blood glucose levels has been studied to a much lesser extent so far [18].

In our study, we first computed theoretical binding affinities corresponding to the strengths of protein-ligand interactions. We then gathered medical data that were available in the literature regarding the influence of beta-blockers on glucose homeostasis. Next, we converted both the gut hormone receptors binding affinities and the medical information into drug rankings. Surprisingly, we observed a significant correlation between these two sets of data leading to the conclusion that beta-blockers with the strongest binding affinities exhibited the smallest negative influence on glucose metabolism. We believe that high binding affinities for incretin receptors, plausibly associated with high efficacy leading to the compensating incretin effect, could be helpful in avoiding or at least decreasing the incidence of drug-induced T2DM while treating hypertension with beta-blockers. Based on the results described here, we developed a web service named GUT-DOCK (http://gut-dock.miningmembrane.com) to propose drug-like molecules from a user’s defined set of compounds that bind strongly to incretin receptors. Assuming that the efficacy of these compounds is experimentally confirmed, e.g. by *in vitro* assays [19], the nominated compounds would likely be the least harmful to glucose metabolism due to the compensating incretin effect. In other words, the user can predict the probability of the off-target drug interactions with the selected GPCRs by computing the theoretical drug-receptor theoretical binding affinity. In addition, GUT-DOCK provides immediate comparison of theoretical binding affinities with corresponding precomputed results obtained for beta-blockers of known diabetogenic effect, which is especially useful when the user’s compound is a beta-blocker. Although GUT-DOCK is the first on-line method for studying off-target interactions of beta-blockers, there are other computational methods that can be used to study associations between new-onset T2DM and, for example, glucocorticosteroids [20]. There are also a few studies on drug target prediction using, e.g., self-organizing maps [21], machine learning algorithms [22] or by performing data-mining of chemogenomic databases [23]. Experimental studies which could provide insights into off-target drug activity are realtively costly and time-consuming, especially in the case of *in-vivo* studies. However, cellular assays and coactivators assays used, e.g., in a recent study about the off-target drug activity for nuclear receptors, seem to be less expensive and reliable tools in this area of research [19]. In addition to the optimization of pharmacotherapy for T2DM treatment, GUT-DOCK can also be used to design novel active pharmaceutical ingredients (API) which demonstrate agonist/antagonist activity when bound to either orthosteric or allosteric binding sites in the transmembrane domains of GCGR, GIPR, GLP1R, VIPR1 and PAC1R receptors. With partial success limited by the performance of Autodock VINA [24], it can also be used in the docking of short peptides to orthosteric binding sites that, in class B GPCRs, are targeted by endogenous hormones.

## Methods

### GPCR models building

To date, the available crystal structures of class B GPCRs (see Table 1) include the N-terminal helix, which is longer than of class A GPCRs, a linker (stalk) joining the transmembrane domain (TMD) and the extracellular domain (ECD) that binds endogenous peptides. There are two possible ligand binding sites: the orthosteric peptide binding site surrounded by extracellular loops and the additional allosteric binding site located between TMH6 and TMH7 that faces the lipid bilayer [25]. The major determinant of the negative (NAM) or positive (PAM) allosteric modulation selectivity for gut hormone receptors is F6.36 (GCGR) [25], which can be substituted with Cys (GLP1R), Leu (GIPR, PAC1R) or Ser (VIPR1). Of the limited GCGR (unbound), GCGR (ligand-bound) and GLP1RGLP1R crystal structures we selected three (PDB id: 4L6R, 5XEZ and 5VEW, respectively) that represent the inactive conformations of the receptors. The latter two structures represent complexes with negative allosteric modulators (NAM) of GCGR and GLP1R: NNC0640 and PF-06372222, respectively.

**Table 1:** Crystal structures of gut hormone receptors [25–28] and endogenous peptides used for receptor model building and model quality assessment.

Receptor	Domain/ligand	Conformational state	PDB id	PDB structure modifications	GPCR models built on this template
GCGR	TMD	Inactive	4L6R	MD (only for GCGR)	GCGR, GIPR, GLP1RGLP1R, VIPR1, PAC1RPAC1R
GCGR	TMD	Inactive	5XEZ	MD (only for GCGR)	GCGR, VIPR1, PAC1RPAC1R
GCGR	TMD, ECD, Glucagon	Active	MD simulation based on 4L6R and 4ERS	Seven N-terminal residues of glucagon truncated	Filtering based on steric hindrances—GCGR, GIPR, GLP1R, VIPR1, PAC1R
GLP1R	TMD	Inactive	5VEW	MD (only for GLP1R)	GLP1R, VIPR1, PAC1R
GLP1R	ECD, GLP	Active	3IOL	Three N-terminal residues of GLP were truncated	Filtering based on steric hindrances—GLP1R
GIPR	ECD, GIP	Active	2QKH	Last six residues of GIP were truncated	Filtering based on steric hindrances—GIPR
VIPR1	VIP	Active	2RRI	None	Filtering based on steric hindrances—VIPR1
VIPR1	ECD	Active	2JOD	None	Filtering based on steric hindrances—VIPR1, PAC1R
PAC1R	PAC-1	Active	2D2P	None	Filtering based on steric hindrances—PAC1R

**Fig 1:**
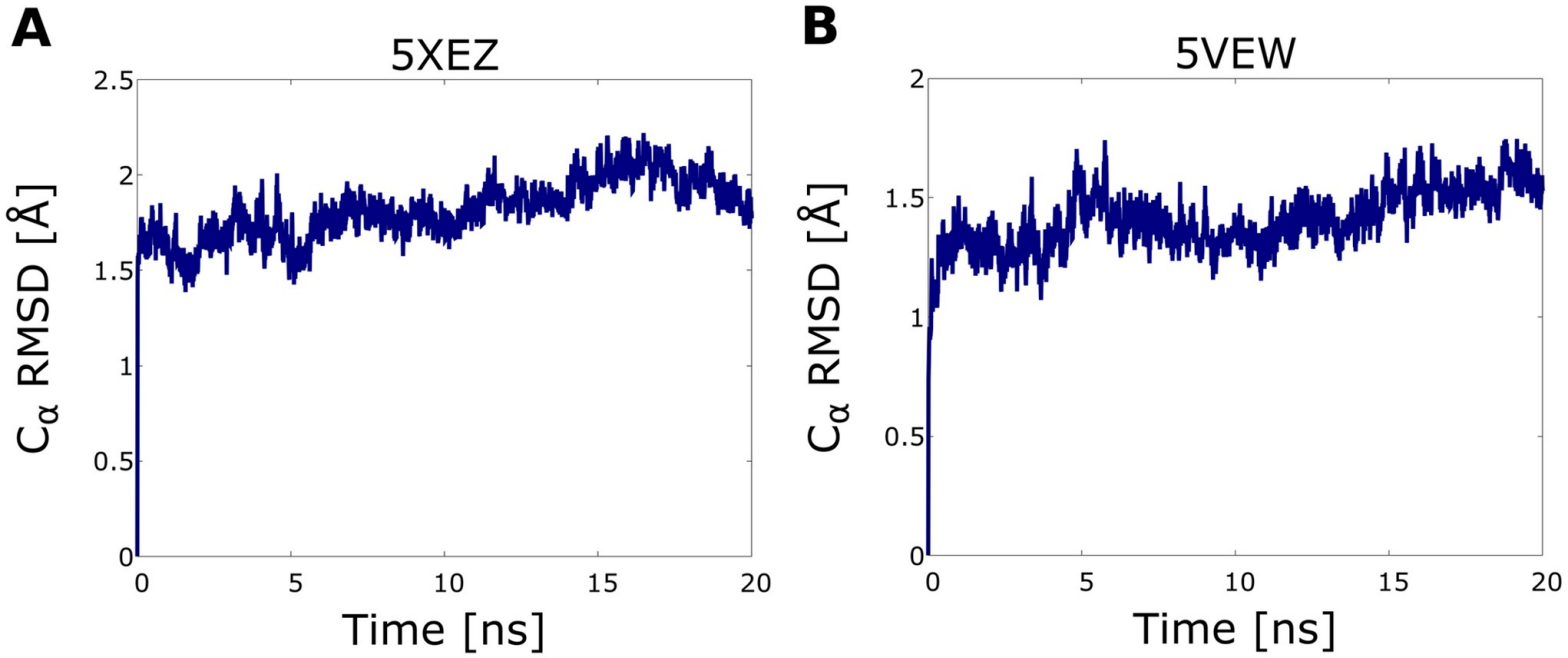
Root mean square deviation curves of the transmembrane helical cores of crystal structures collected in equilibrium MD simulations. The respective PDB id of GCGR and GLP1R receptors: 5XEZ (a) and 5VEW (b).

The GPCR models used in this study were either built via homology modeling using the available class B GPCR crystal structures and our previously developed web service GPCRM [29, 30] or prepared via molecular dynamics simulations starting from crystal structures (see Table 1). In the first case, a total number of 3000 models for each template/receptor pair was generated. The most suitable template structure was selected based on sequence identity with the target sequence (see Table B in S1 File). 1500 models were discarded based on steric hindrance scores (see **GPCR models filtering based on steric hindrances**). The remaining 1500 models were clustered using the Rosetta3.5 cluster application and evaluated with BCL::Score using knowledge-based potentials derived specifically for membrane proteins [29]. The five lowest energy models were selected from the five largest clusters for virtual screening (VS) (see **Ligand-based filtering of GPCR models—a virtual screening study**). In the case of GCGR and GLP1R, their crystal structures were used in 20ns MD simulations to generate an ensemble of receptor conformations (see **Molecular dynamics simulations**). Then, 2000 snapshots were recorded for each structure/receptor pair (see Fig 1). A total of 1000 structures were discarded based on the steric hindrances scores. The remaining 1000 structures were clustered and evaluated with BCL::Score. The five lowest energy structures were selected from the five largest clusters for VS. Based on the enrichment data (see Table 2), the best model for each receptor was selected for further analysis.

**Table 2:** The enrichment data for gut hormone receptors.

Receptor	Template / Binding site / Model number	BEDROC(alpha = 20)	ROC	AUC	EF1%	EF5%	EF10%
GLP1R	5VEW—allosteric						
	Model 1	0.589	0.94	0.94	29	14	7
	Model 2	0.559	0.98	0.97	38	10	8
	5VEW—orthosteric						
	Model 1	0.423	0.93	0.92	0	12	6
	Model 2	0.330	0.93	0.92	9.6	6.1	5
GIPR	4L6R –orthosteric						
	Model 1	0.773	0.94	0.93	50	18	8.9
	Model 2	0.668	0.95	0.95	40	14	7
	4L6R –allosteric						
	Model 1	0.453	0.94	0.94	10	12	8.9
	Model 2	0.518	0.92	0.91	30	10	7
GCGR	5XEZ—orthosteric						
	Model 1	0.741	0.91	0.91	43	16	8
	Model 2	0.440	0.89	0.89	11	10	6
	5XEZ—allosteric						
	Model 1	0.441	0.90	0.89	0	12	8
	Model 2	0.419	0.89	0.88	11	10	7
Results for corresponding X-ray structures							
Receptor	PDB id / Binding site	BEDROC(alpha = 20)	ROC	AUC	EF1%	EF5%	EF10%
GLP1R	5VEW—orthosteric	0.441	0.90	0.90	9.6	10	7
	5VEW—allosteric	0.278	0.87	0.87	0	6.1	6
GCGR	5XEZ—orthosteric	0.197	0.77	0.77	0	4	4
	5XEZ—allosteric	0.678	0.92	0.91	43	14	8

### Molecular dynamics simulations

We performed short, 20 ns molecular dynamics simulations to generate the ensemble of receptor conformations based on the GLP1R and GCGR crystal structures. Each system, containing the protein embedded within the membrane and solvated, was prepared using the CHARMM-GUI Membrane Builder (http://www.charmm-gui.org) [31–34]. The membrane consisted of POPC and cholesterol molecules in a proportion of 5:1. In each case, the system was neutralized by the addition of Na^+^ and Cl^-^ ions, with an ionic concentration of 0.15 M. The size of the box was set so that its boundaries were at least 15 Å away from the protein atoms. Each of the generated systems contained approximately 100 POPC and 20 cholesterol molecules, and the total number of atoms ranged from 47213 (5VEW) to 69309 (5XEZ). The applied force field was Charmm36 [35]. Next, each system was minimized with 2500 steps of the steepest descent minimization followed by 2500 steps of conjugate gradient minimization. During minimization, position restraints were applied to all atoms of the protein, with a force constant of 10.0 kcal mol^-1^ Å^-2^. The positions P atoms in POPC molecules and O3 atoms in cholesterol molecules were restrained with a force constant of 2.5 kcal mol^-1^ Å^-2^. In the subsequent six consecutive short runs of the equilibration process, the position constraints were gradually decreased, firstly in two runs of 100 ps in the NVT ensemble, and then in four runs of 100 ps in the NPT ensemble. Temperature was controlled by the Langevin thermostat, with a friction coefficient of 1.0 ps^-1^, and a value of 310 K. The external pressure was 1 bar controlled by the Berendsen barostat. The time integration step was set to 0.001 ps during the first equilibrium runs and set to 0.002 ps thereafter. The SHAKE algorithm was turned on to constrain bonds involving hydrogen atoms. Finally, an unconstrained 20 ns-long production run was performed for each system. All molecular dynamics simulations were performed with the GPU version of the pmemd module of the AMBER14 package [36]. During production runs, conformational fluctuations stabilized after about 10 ns (see Fig 1). The RMSD value for the transmembrane region of GCRG was equal to approximately 2.0 Å and that of GLP1R RMSD value was approximately 1.5 Å. RMSD values were higher, approximately 2.2–3.3 Å for the whole TMD due to the mobility of loops. Other observed structural changes could be caused by the fact, that ligands, present in crystal structures, were not included in the MD simulations.

### GPCR models filtering based on steric hindrances

To assess the accuracy of the modeled external loops of the studied receptors, we applied the following procedure. We defined a three-dimensional Cartesian grid which covered the orthosteric binding site occupied by the endogenous peptide. The spacing between the grid vertices was the same in all directions and was equal to 1 Å. Each of the heavy atoms of these endogenous peptides was assigned to the proper unit cell based on its coordinates, and each of these atoms was treated with the same weight. In this way we calculated the ligand density, which was proportional to the number of ligand atoms within each grid cell for a given set of ligand peptides. The calculated ligand density was then applied to the analysis of the binding pockets of G-protein coupled receptors, the structures of which were predicted using homology modeling. In this way, it was possible to distinguish receptor models with extracellular loops excessively occupying the orthosteric binding site. The ligand density grid was divided into the following three areas. The first area covered the space of grid points assigned with nonzero density values. The second area consisted of the space of grid points with the ligand density equal to zero but with the nonzero neighboring grid points. This area formed a shell surrounding the non-zero ligand density grid point space. The first area was suitable for the assessment of steric hindrances caused by a peptide, while the second area was useful in the case of small-molecule ligands (data not shown). The final steric hindrance score for a peptide and a given homology model of a receptor was equal to the number of nonzero ligand density grid points covered by the receptor (TMD and loops).

### Ligand-based filtering of GPCR models—A virtual screening study

Models of gut hormone receptors were generated using: crystal template structures, GPCRM, model quality assessment with the BCL::Score scoring function [37], Rosetta3.5 clustering, filtering based on steric hindrances and MD simulations. These models, after subjecting to the ‘Prepare Protein’ procedure in Glide were evaluated using the virtual screening (VS) procedure to retrieve the best-performing decoys [38]. For VS, we used a set of 10 known active ligands for each receptor (see Table A in S1 File) and 500 decoys (50 for every ligand) generated using DUD-E [39]. Known actives were retrieved from the BindingDB [40] and PubChem [41] databases. The VS procedure was carried out using SP-Glide (Schrodinger) [42]. The binding sites, orthosteric and allosteric, were treated separately. Based on the current knowledge, we could not eliminate any of the possible binding sites of the tested receptors as potential off-target interaction sites. To select the best-performing in VS we used typical metrics used for the evaluation of VS results: EF1%, EF5%, EF10%, ROC, AUC and BEDROC(alpha = 20) [38, 43]. In Table 2, we presented the results of the two best-performing models for each receptor and each binding site. Of these two models, one model for each receptor one was selected to be used in GUT-DOCK.

In Table 2, we also present results of the enrichment study including corresponding crystal structures of GLP1R and GCGR receptors (PDB id: 5VEW and 5XEZ, respectively). Crystal structures of both receptors performed slightly worse in comparison to our MD-refined models. The performance of crystal structures, homology models and MD-refined models in virtual screening was discussed in details previously, e.g., in [29, 44]. The current study only confirmed that the superiority of crystal structures in VS should not be treated as a rule.

### GUT-DOCK—The description of the web service pipeline

**Fig 2:**
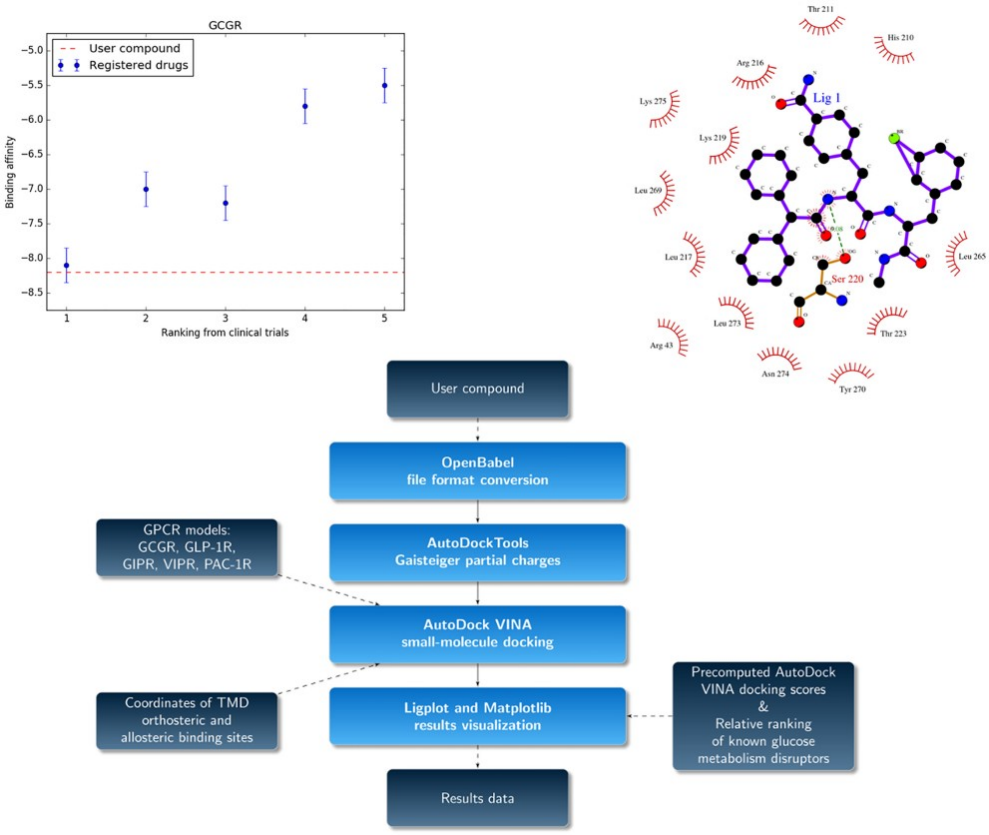
The graphical interface and algorithm of GUT-DOCK. Top panels—example GUT-DOCK results for compound 15 docked to GCGR. A bottom panel—the schematic pipeline of GUT-DOCK.

GPCR models of five class B receptors generated with the procedure described above were implemented in GUT-DOCK to serve for docking purposes. Before implementing these models in GUT-DOCK we used a standalone version of Autodock Tools to generate pdbqt receptor files. The main core of GUT-DOCK includes the docking of user-provided small-molecule ligands to preprepared GPCR models (see Fig 2). Those models were validated in the VS procedure described above. There are two binding sites (orthosteric and allosteric) in each receptor that can be targeted by docking. The docking procedure is carried out by Autodock—VINA [45]. Here, the original Autodock VINA scoring function [45] was used to assess ligand poses obtained from the flexible ligand-rigid receptor docking. No modifications were made to the original Autodock VINA program or to its scoring function. Thus, we did not include any benchmarking analysis here. Autodock VINA has been tested and benchmarked with other docking programs in many previous studies with a highly positive result confirmed by the number of citations of the original manuscript [45]. Autodock VINA was also implemented in other web services for small-molecule docking [46–48]. The user-provided ligand is converted into a pdb file with Open Babel ver. 2.3.2 [49] and into a pdbqt file including Gasteiger charges using AutoDockTools ver. 1.5.7. A single top-scoring ligand pose for each receptor is presented as a web service output. Ligplot ver. 4.0 [50] is used to depict ligand-receptor interactions and crucial binding residues inside the binding site. To generate Matplotlib plots (see an example in Fig 2) we used the T2DM-related ranking of several selected beta-blockers (nebivolol, carvedilol, labetalol, atenolol and metoprolol, see Table 3) and the respective precomputed Autodock VINA scores obtained from docking these beta-blockers to a GPCR receptor model. A user-provided compound is assigned no rank (see Fig 2); however, provided the compound is a beta-blocker or a molecule with a similar mechanism of action, one can predict its relative rank by comparing its docking score with precomputed docking scores and known ranks provided for beta-blockers. Regarding the selected test set of beta-blockers, we selected only a few well-known beta-blockers for which the complete medical information for hyperglycemia cases could be found in the literature. Consequently, we could easily construct their relative ranking with respect to new-onset diabetes risk during treatment. Notably, the medical information on drug side effects in the literature is sparse and often contradictory and should be used with caution. In our second manuscript [4], we address that problem using statins as an example.

**Table 3:** Drug-induced T2DM risk ranking of beta-blockers based on medical information from [58, 61–66] and GUT-DOCK results.

Name	Rank based on medical information	Rank based on medical information combined with GUT-DOCK results
compound 15	No data available yet	0
nebivolol*	1	1
carvedilol	1	2
labetalol	2	3
atenolol	3	4
metoprolol	4	5

* In a very recent clinical trial [67] carvedilol and nebivolol was assigned similar ranks, yet we believe that additional medical information should be gathered on that matter in the future.

The GUT-DOCK web service is divided into two parts. The first part is a responsive user interface that can run queries and display results. The user interface is a web interface that was constructed using HTML5 with jQuery, jQuery UI, Lightbox, Bootstrap libraries. The web interface was designed to fit both desktop and mobile devices. The web service itself was written in Python using the Django Framework for serving the web application and dynamically generated HTML5 pages for managing the MySQL database. The second part is dedicated to computing user jobs independently from the user’s web actions. A link to the results is sent by email when the job is completed. The computational server runs under Linux on a multi-core CPU and that allows for the Autodock VINA multithreading. The user can run multiple jobs at the same time depending on current computational resources.

In GUT-DOCK and in VS we assumed that docking scores obtained with Autodock VINA and Glide correlated with the binding affinities. This assumption was based on the observed high success rates of virtual screening comparing experimental screening (see the accompanying manuscript [4]). Although there are exceptions to this assumption [51] which confirms the necessity for the continuous benchmarking of docking programs [39] many studies confirmed such statement [45, 52–54]. Nevertheless, experimental studies are certainly needed to confirm whether the observed high affinity for the selected receptor of a given drug is indeed associated with its high efficacy and alters the receptor function [55].

## Results and discussion

**Fig 3:**
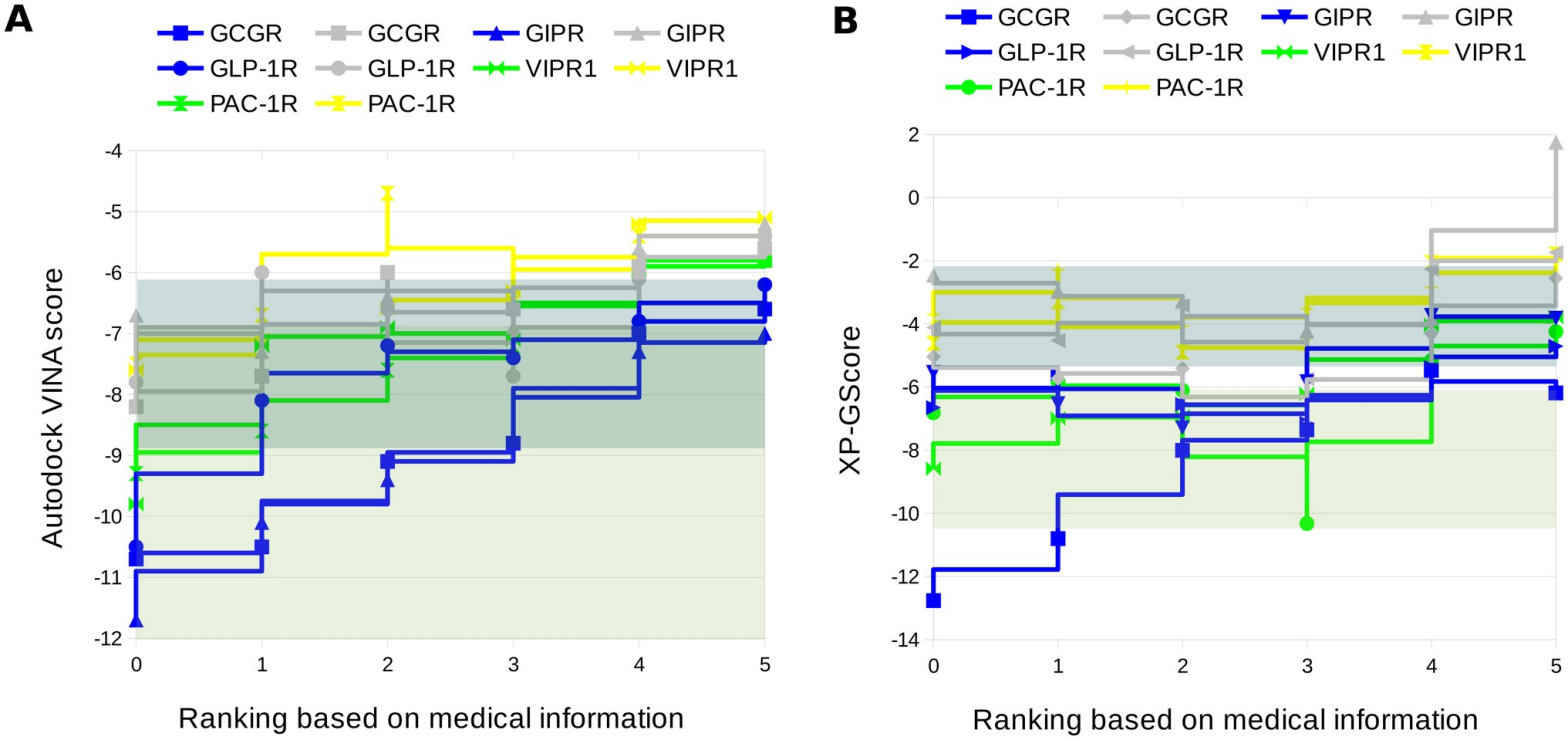
Beta-blocker-induced diabetes risk vs. the binding affinity for incretin receptors and other class B GPCRs. GUT-DOCK (Autodock VINA) results for selected beta-blockers (A). T2DM-related ranks were assigned according to [58, 61–66] and average GUT-DOCK results (see Table 3): 0 –compound 15, 1 –carvedilol, 2 –nebivolol, 3 –labetalol, 4—atenolol, 5 –metoprolol (1 –the least disturbing beta-blocker, 0 –no clinical data). The orthosteric binding site is represented by blue and green points and lines in a stepped chart, allosteric by light grey and yellow points and lines. Here, we added trend lines only for the sake of the readability of the plot. (B) Glide-derived binding affinities of commonly used beta-blockers towards the selected class B GPCRs vs. the drug ranking based on T2DM-oriented clinical trials. Assigned T2DM-related ranks—the same as in (A). Grey (orthosteric) and green (allosteric) transparent boxes indicated the values ranges of binding affinities computed with Autodock VINA and glide for active ligands of GCGR (see Table A in S1 File).

It was suggested in [56] that beta-1 selective beta-blockers exhibit fewer side effects due to differences between the expression patterns of beta-1 (mostly heart and kidney) and beta-2-adrenergic (heart, kidney, gastrointestinal tract and liver) receptors. However, another study [57] proved that selective beta-1-blockers elevated fasting blood glucose. In our study only the newer, vasodilating but not necessarily beta-1 selective, third generation beta-blockers (carvedilol and nebivolol) exhibited an increased binding affinity for gut hormone GPCRs (see Fig 3), which might be beneficial for diabetic patients. This result was also confirmed in clinical trials [58]. Binding affinities of beta-blockers for GCGR were in the similar values range as binding affinities computed for active ligands of this receptor (see ranges in Fig 3) which additionally suggested that beta-blockers might interact with gut hormone receptors. The docking results described above were obtained using Autodock VINA [45]. However, we repeated computations using the licensed software—Glide (Schrodinger). The results were similar using these two programs (see Fig 3), with correlation coefficients slightly higher in the case of Autodock VINA (GCGR, the orthosteric site: 0.934 and the allosteric site: 0.682; GIPR: 0.964 and 0.871; GLP1R: 0.880 and 0.458) than those in Glide (GCGR: 0.793; 0.747; GIPR: 0.942; 0.664; GLP1R: 0.631; 0.674, respectively). Additionally, we computed correlation coefficients for Autodock VINA results obtained for VIPR1 and PAC1R: 0.912 (orthosteric) and 0.951 (allosteric) and 0.920 (orthosteric) and 0.215 (allosteric), respectively. The corresponding coefficients for the Glide results were 0.967 and 0.793 for VIPR1 and 0.377 and 0.567 for PAC1R, respectively. The correlation coefficients were lower for PAC1R than for the incretin receptors, at least in the case of one (orthosteric or allosteric) binding site. However, in the case of VIPR1, with a sequence more similar to incretin receptors sequences than PAC1R (see Table B in S1 File), correlation coefficients were as high as those of incretin receptors. The docking scores for Autodock VINA and Glide are based on different scoring functions but both of them were successfully used in VS studies [42, 59, 60]. To compute the above correlation coefficients, we used the beta-blockers’ ranking generated using the medical information (see Table 3). By comparing theoretical binding affinities towards incretin receptors with clinical data on beta-blocker-induced T2DM risk rates and computing the respective correlation coefficients, we could partially indirectly test the validity of the assumptions stated in the **Introduction**. Indeed, high correlation coefficients for gut hormone receptors confirmed that the compensating incretin effect could be, at least partially, responsible for the decreased risk of hyperglycemia associated with the new generation of beta-blockers. On the other hand, the weak binding affinities of the previous generation of beta-blockers for gut hormone receptors could indicate that their chemical structures do not fit the receptor binding sites, and thus the compensating incretin effect could not be enhanced.

As we mentioned above, allosteric and orthosteric binding sites slightly differed in the strength of drug-receptor interactions. In almost all cases, ligands were bound more strongly to the more spacious orthosteric site than to the allosteric site. To date, all available crystal structure of class B GPCRs contain only antagonists or negative allosteric modulators, and no orthosteric nonpeptide small-molecule ligands mimicking the mechanism of action of the endogenous peptide have been reported in the PDB. In contrast, analogs of endogenous peptides have been reported (see, e.g., the recently released crystal structure of GCGR with the partial peptidic agonist NNC1702 [68]). Hollenstein et al.[69] reported that the GCGR orthosteric site fitted to peptides is open and occupied by a bulk-like solvent with only a single ‘druggable’ hotspot at the bottom and thus is weakly ‘druggable’ for small, nonpeptide molecules.

**Fig 4:**
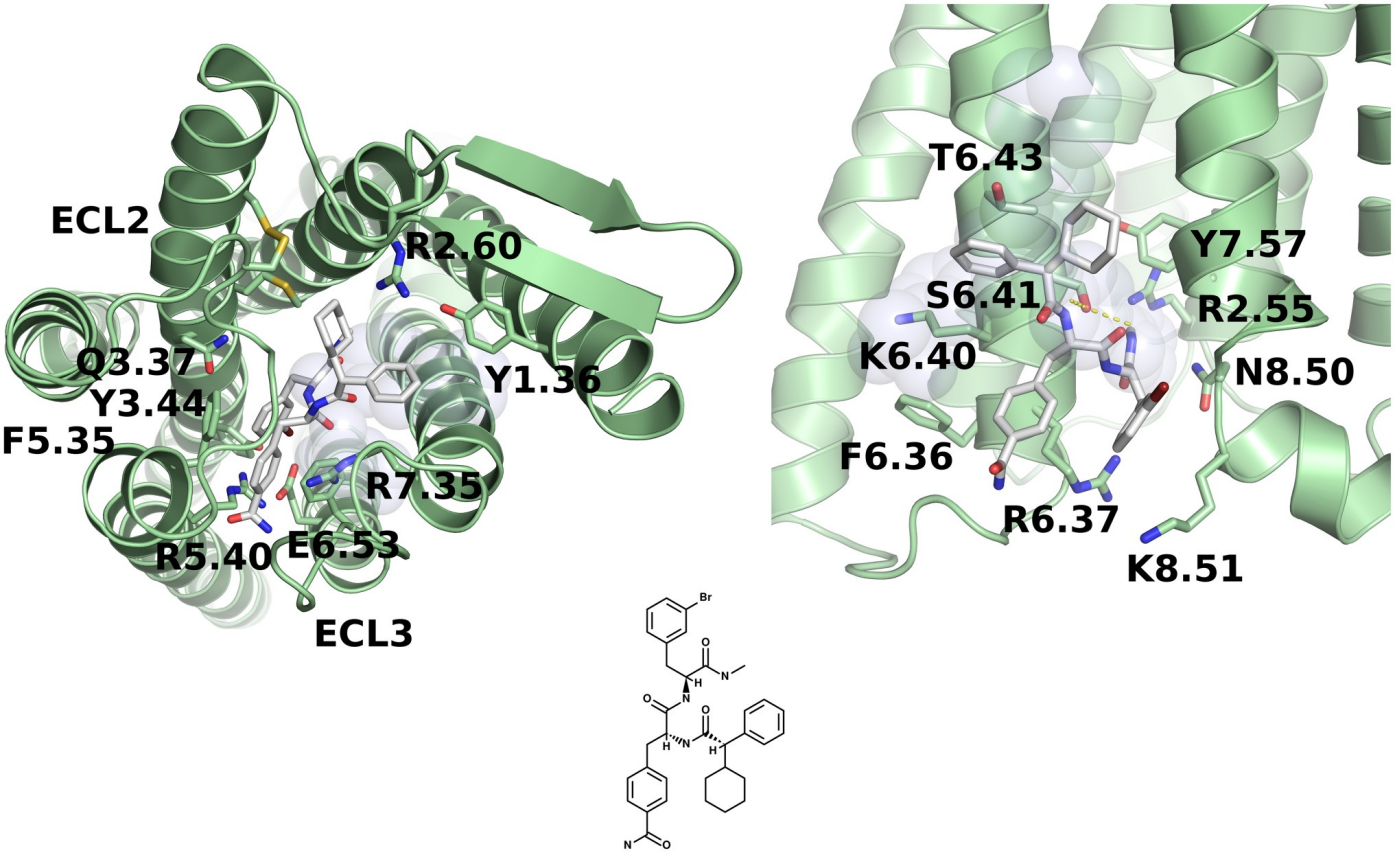
GUT-DOCK results for the docking of compound 15 to GCGR. (A)—the orthosteric binding site (the Autodock VINA docking score equal to -10.7), (B)–the allosteric site (the Autodock VINA docking score equal to -8.2). Here, polar contacts are depicted as yellow dashed lines and residues involved in binding are numbered using the class B GPCR Wooten scheme [74].

In general, if a drug binds to an orthosteric GPCR site, it may block that binding site from its endogenous ligand and thus may demonstrate an inhibitory (antagonistic) effect on the receptor signaling pathway. Nevertheless, it may also act as an agonist, activating the receptor similarly to the endogenous ligand. In the case of GCGR, the former effect would be highly desirable due to the inactivation of the diabetogenic mechanism associated with glucagon [70]. Allosteric GPCR sites can be targeted by either positive or negative receptor signaling modulators. Positive allosteric modulators (PAMs) of GIPR and GLP1R evoke the incretin effect and thus regulate glucose homeostasis. Potential inhibition of GCGR and/or positive modulation of GLP1R and GIPR signaling pathways would represent a beneficial side effect of a given drug. However, our study did not include both the active and inactive conformations of each receptor and thus we could not unambiguously determine what effect a tested ligand could demonstrate on the receptor signaling pathway. We added a blind test to our study including a recently discovered beta-blocker from a DNA-encoded small molecule library [71]: compound 15. In contrast to all known beta-blockers targeting the orthosteric binding site of the beta-2 adrenergic receptor, compound 15 is a negative allosteric modulator (NAM) of that receptor. In this blind test, we aimed to determine if that newly discovered beta-blocker could be useful in the treatment of heart failure and/or hypertension in diabetic patients due to the compensating incretin effect. In Fig 2, we presented an example screenshot of the GUT-DOCK results web page for that compound. Here, we selected GCGR as a GPCR receptor. The docking results for compound 15 were represented by a red dashed line, while points indicated results for other beta-blockers with known effect on glucose homeostasis (see Table 3). The error bars for Autodock VINA scores were computed based on the observed distribution of results for compound 15. In Fig 4, we presented the most plausible binding mode of compound 15 to GCGR. Most class B GPCR conserved residues [69] are in contact with the ligand, especially S6.41 (a hydrogen bond), R6.37 and N8.50, which are located in the allosteric site. A similar binding mode of compound 15 was observed also in the case of GLP1R (see Figures A-C in S1 File).

Compound 15 demonstrated similar binding affinities for gut hormone receptors similar to carvedilol and nebivolol (see Fig 3). In almost all cases, independently of the type of the binding site (orthosteric vs. allosteric), compound 15 was ranked by Autodock VINA as the best or the second-best ligand out of all tested beta-blockers. This finding suggested that means that compound 15 may enhance the incretin effect and thus may demonstrate satisfactory results in phase II clinical trials when the new-onset diabetes risk is assessed during treatment of hypertension (or heart failure).

It is noteworthy that the computational engine of GUT-DOCK is not a novel one. We simply implemented the well-known programs and methods for small-molecule docking that were tested and assessed elsewhere (see [45] for Autodock VINA and [49] for OpenBabel). For this reason we did not perform detailed tests of our web service in the current study. The testing of Autdock VINA performance in small-molecule docking to GPCRs, especially to the class B GPCRs, requires a separate study that we hope to carry out in future.

Nevertheless, for the sake of the current study, we decided to test whether Autodock VINA is able to reproduce the ligand binding modes observed in crystal structures of gut hormone receptors. We performed a short, self-docking study for GCGR and GLP1R receptors using our web service. We used ligands from the GLP1R and GCGR crystal structures (PDB id: 5VEW and 5XEZ, respectively) that were extracted as three-dimensional structures deposited as SDF files in the PubChem Database [41]. In the case of GLP1R, the best pose generated with Autodock VINA using the specific MD-refined receptor structure, docking box coordinates, box size and other settings in GUT-DOCK was ranked 6, and the heavy atom RMSD with respect to the crystal ligand orientation was 4.73 Å. The RMSD of the top-ranked pose according to the Autodock VINA scoring function was 9.34 Å. Most importantly, the Autodock VINA score for that ligand was the best (the lowest value) in the case of docking to the GLP1R structure (-8.2) while docking to other class B GPCRs resulted in ligand poses with worse scores (GCGR: -7.0; GIPR: -6.5; VIPR1: -8.0; PAC1R: -6.8). This finding confirms that Autodock VINA is able to accurately predict the best receptor (with the highest affinity) for the given ligand. This result was crucial for the current study in which we tried to assess whether the given ligand (beta-blocker) demonstrated binding affinity for any of the selected class B GPCRs and, if the answer was ‘yes’, for which GPCR the binding affinity was the highest.

**Fig 5:**
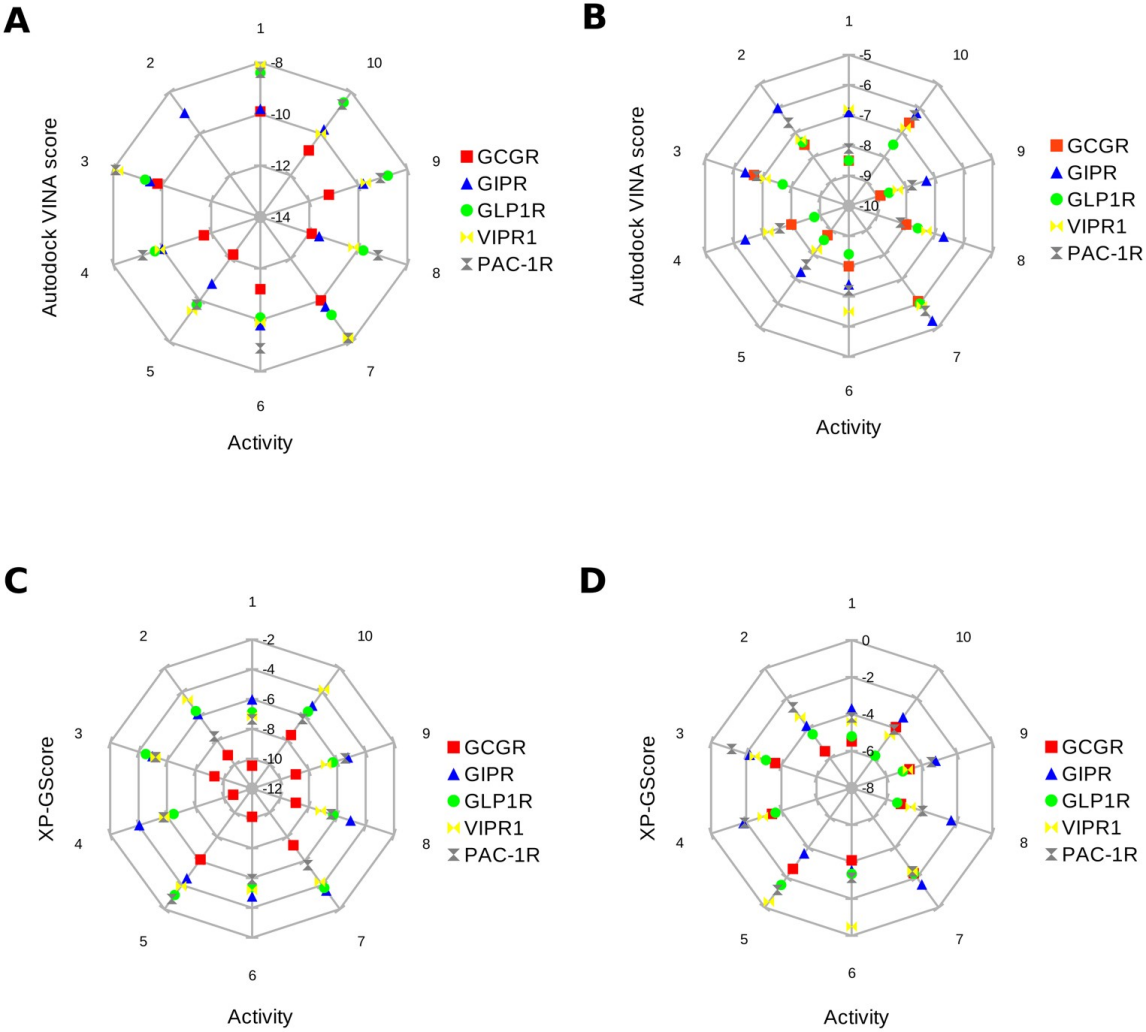
Radar charts (spider plots) illustrating the Autodock VINA and Glide abilities for recognition of the best target protein for a given ligand. Here, we used 10 active ligands of GCGR (see Table A in S1 File) sorted with respect to their activity (inhibitor constant). Left plots (A and C) represent results for the orthosteric sites while plots on the right (B and D) represent results for the allosteric sites.

The results of the self-docking study for GCGR were less worse than for GLP1R. The crystal orientation of the GCGR ligand was not very accurately predicted (RMSD of the best pose ranked as third: 7.27; RMSD of the first ranked pose: 9.63). However, also in this case, the best receptor for the given ligand was accurately predicted based on the Autodock VINA scoring (GCGR: -8.9; GIPR: -8.7; GLP1R: -8.6; VIPR1: -8.0; PAC1R: -7.6). We believe that the results, for both GLP1R and GCGR confirmed the usefulness of Autodock VINA for the prediction of the best GPCR receptor (with the highest binding affinity) for a given ligand, even if the binding mode was not accurate. However, a more detailed study could be carried out on this subject in the future. Radar charts presented in Fig 5 are the additional confirmation of the ability of GUT-DOCK to select the best protein target for a given compound. In case of both binding sites (orthosteric better), active ligands for GCGR (see Table A in S1 File) exhibited the best docking scores when bound to GCGR and the worst scores when bound to non-incretin receptors (VIPR1 and PAC1R). Interestingly, docking scores obtained in the case of GIPR were on the similar level like in the case of VIPR1 and PAC1R. However, we believe that it could be a result of the fact that the GIPR homology model was built using an older unliganded template structure of GCGR (PDB ID: 4L6R) instead of a newer template structure (PDB ID: 5XEZ).

## Conclusions

In our study, we hypothesized that off-target interactions of specificdrugs with gut hormone receptors GLP1R, GIPR and GCGR could be a way to compensate for the negative influence of each on glucose homeostasis leading to drug-induced diabetes. For such off-target interactions to be beneficial, they should result in the enhancement of the incretin effect, e.g., when a drug acts as PAM or an agonist of GIPR and GLP1R and NAM or an inhibitor of GCGR. Thus, improvements in insulin secretion and normal glucose serum levels could be observed. In contrast, the inhibition of GLP1R increases blood glucose levels [72], and agonistic or PAM effects on GCGR may unfavorably increase glucagon serum levels [15]. In our study, which was focused on beta-blockers, we assessed the probabilities of the off-target interactions of beta-blockers with gut hormone receptors regardless of their specific effect (stimulating or inhibiting) on signal transduction. We analyzed theoretical binding affinities provided by two different docking programs, yet our observations should certainly be confirmed with experimental studies. Nevertheless, experimental studies in this case may require long-term observations of patients in clinical conditions.

The current study on beta-blockers and a second study [4] describing other diabetes-inducing drug classes (statins and steroids) are focused on the quantitative description of potential off-target interactions without detailed qualitative descriptions of ligand binding modes, which could provide additional insight into structure-activity relationships. However the molecular mechanisms of the unintended drug-target interactions, including the relationship between ligand binding affinities and efficacies in comparison with the intended drug-target interactions, are still not fully understood in detail. There are only a few experimental studies in this field [73], yet these do not involve beta-blockers and gut hormone receptors.

Our work is an example of a novel computational approach to the fast and low-cost prediction of the off-target drug interactions with class B GPCRs. Such information on the enhancement of the compensating incretin effect is especially important for diabetic patients. However, we did not intend for this study to be the only guidance for modification of pharmacotherapies for DM, as there are many other side effects (see http://sideeffects.embl.de), e. g., angiopathy, that should be taken into account while optimizing pharmacotherapy for diabetic patients.

A freely available web service developed during this study (GUT-DOCK) can also be used to discover new therapeutics and to predict off-target interactions of any small molecule or small peptide with class B GPCRs. Easy-to-download GPCR models can also be used in further drug discovery studies.

## Supporting information

S1 File Supplementary material. A file including: active compounds lists, sequence identity between templates and targets from the current study, figures presenting GUT-DOCK results for compound 15 and GLP1R.
(PDF)

S2 File A file including data generated during the current study. (XLSX)

## Abbreviations

ADME: absorption, distribution, metabolism, and excretion
DM: diabetes mellitus
ECD: extracellular domain
GCGR: glucagon receptor
GIPR: gastric inhibitory polypeptide receptor
GLP1R: glucagon-like peptide-1 receptor
GPCR: G protein-coupled receptor
MD: molecular dynamics
PAC1R: pituitary adenylate cyclase-activating polypeptide 1 receptor
T2DM: type 2 diabetes mellitus
TMD: transmembrane domain
VIPR1: vasoactive intestinal polypeptide receptor 1
VS: virtual screening
