# Supplementary material for: Potential off-target effects of beta-blockers on gut hormone receptors: In silico study including GUT-DOCK—A web service for small-molecule docking
Source: PLoS One. 2019 Jan 25;14(1):e0210705. doi: 10.1371/journal.pone.0210705 (PMC6347188; doi:10.1371/journal.pone.0210705)

Potential off-target effects of beta-blockers on gut hormone receptors: in silico study including GUT-DOCK – a web service for small-molecule docking

Pawel Pasznik, Ewelina Rutkowska, Szymon Niewieczerzal, Judyta Cielecka-Piontek, Dorota Latek

Supplementary material

A file including: active compounds lists, sequence identity between templates and targets from the current study, figures presenting GUT-DOCK results for compound 15 and GLP1R.

Table A. Active compounds used for the enrichment computations.

Receptor	CHEMBL ID	SMILES	Inhibitor constant [micromolar][1]
GCGR	CHEMBL3673123	C1=CC=C(C(=C1)CNC2=CC=C(C=C2)C3=CC=C(C=C3)Cl)C4=CC=C(C=C4)C(=O)NCCC(=O)O	0.06
	CHEMBL1933354	CCCC(C1=CC=C(C=C1)C(=O)NCCC(=O)O)OC2=CN(N=C2)C3=CC=CC(=C3)CC	4.53
	CHEMBL1933348	CCCC1=C(C(=C(C=C1C(C)C)C(C)C)C(C)O)C2=CC=C(C=C2)F	0.078
	CHEMBL1933361	CCCC(C1=CC=C(C=C1)C(=O)NCCC(=O)O)OC2=CN(N=C2)C3=NC=C(C=C3)C(F)(F)F	16.4
	CHEMBL3799802	CC(C1=CC=C(C=C1)C(=O)NCCC(=O)O)N2C(=CC(=N2)C3=CC(=CC(=C3)Cl)Cl)C4=CC5=C(C=C4)C=C(C=C5)OC	0.1
	CHEMBL486634	C1CCC(=CC1)C2=CC=C(C=C2)N(CC3=CC=C(C=C3)C(=O)NCC(C(=O)O)O)C(=O)NC4=CC(=CC(=C4)Cl)Cl	0.014[2]
	CHEMBL1644183	C1=CC(=CC=C1CN2C(=CC(=N2)C3=CC(=CC(=C3)Cl)Cl)C4=CC=C(C=C4)OC(F)(F)F)C(=O)NCCC(=O)O	0.044
	CHEMBL1644178	CC(C)(C)C1CCC(CC1)C2=CC(=NN2CC3=CC=C(C=C3)C(=O)NC4=NNN=N4)C5=CC=C(C=C5)OC(F)(F)F	0.17
	CHEMBL1644180	C1=CC(=CC=C1CN2C(=CC(=N2)C3=CC=C(C=C3)OC(F)(F)F)C4=CC=C(C=C4)OC(F)(F)F)C(=O)NC5=NNN=N5	0.047
	CHEMBL1933358	CCCC(C1=CC=C(C=C1)C(=O)NCCC(=O)O)OC2=CN(N=C2)C3=CC(=CC=C3)OC	19.1
GIPR	CHEMBL446821	C1CC2=C(C1)C=C(C=C2)N(CC3=CC=C(C=C3)C(=O)NCCC(=O)O)C4=NC(=CS4)C5=CC=C(C=C5)OC(F)(F)F	
	CHEMBL501628	C1=CC(=CC=C1CN(C2=CC=C(C=C2)OC(F)(F)F)C3=NC(=CS3)C4=CC=C(C=C4)C(F)(F)F)C(=O)NCCC(=O)O	
	CHEMBL62444	CC(C)(C)C1CCC(CC1)N(CC2=CC=C(C=C2)C(=O)NCCC(=O)O)C(=O)NC3=CC=C(C=C3)OC(F)(F)F	
	CHEMBL232224	CC(C)(C)C1CCC(CC1)N(C2CCC3=C2C=CC(=C3)C(=O)NCCC(=O)O)C(=O)CC4	

		=CC=C(C=C4)OC(F)(F)F
	CHEMBL411832	CC(C)(C)C1CCC(CC1)N(C2CCOC3=C2C=CC(=C3)C(=O)NC4=NNN=N4)C(=O)NC5=CC=C(C=C5)OC(F)(F)F
	CHEMBL452067	CC1=CC(=CC(=C1)NC(=O)N(CC2=CC=C(C=C2)C(=O)NCC(C(=O)O)O)C3CCC(CC3)C(C)(C)C)C
	CHEMBL452311	C1CCC(CC1)C2=CC=C(C=C2)N(CC3=CC=C(C=C3)C(=O)NCC(C(=O)O)O)C(=O)NC4=CC(=CC=C4)Br
	CHEMBL456738	COC1=CC(=CC(=C1)NC(=O)N(CC2=CC=C(C=C2)C(=O)NCC(C(=O)O)O)C3=CC=C(C=C3)C4CCCCC4)C(F)(F)F
	CHEMBL453457	C1CCC(CC1)C2=CC=C(C=C2)N(CC3=CC=C(C=C3)C(=O)NCC(C(=O)O)O)C(=O)NC4=CC=C(C=C4)SC(F)(F)F
	CHEMBL1922839	CCCOC1=CC(=CC2=C1N(C(=NC3=CC=C(C=C3)C(C)(C)C)N2CC4=CC=C(C=C4)C(=O)NC5=NNN=N5)C)C(F)(F)F
GLPR1	CHEMBL198736	CC(C)(C)C1CCC(CC1)N(CC2=CC=C(C=C2)C(=O)NCCC(=O)O)C(=O)NC3=CC=C(C=C3)OC(F)(F)F
	CHEMBL219384	CC(C)(C)C1=CC=C(C=C1)N(CC2=CC=C(C=C2)C(=O)NCCC(=O)O)C(=O)NC3=CC=C(C=C3)OC(F)(F)F
	CHEMBL386446	C1CCC(=CC1)C2=CC=C(C=C2)N(CC3=CC=C(C=C3)C(=O)NCCC(=O)O)C(=O)NC4=CC(=CC(=C4)Cl)Cl
	CHEMBL487476	C1CCC(=CC1)C2=CC=C(C=C2)N(CC3=CC=C(C=C3)C(=O)NCC(C(=O)O)O)C(=O)NC4=CC(=CC(=C4)Cl)Cl
	CHEMBL519903	C1CCC(=CC1)C2=CC=C(C=C2)N(CC3=CC=C(C=C3)C(=O)NCC(C(=O)O)O)C(=O)NC4=CC(=CC(=C4)Cl)Cl
	CHEMBL499160	CC(C)(C)C1CCC(CC1)N(CC2=CC=C(C=C2)C(=O)NCC(C(=O)O)O)C(=O)NC3=CC(=CC(=C3)C(F)(F)F)C(F)(F)F
	CHEMBL452310	COC(CNC(=O)C1=CC=C(C=C1)CN(C2=CC=C(C=C2)C3=CCCCC3)C(=O)NC4=CC(=CC(=C4)Cl)Cl)C(=O)O
	CHEMBL1933365	C1CCC(C1)C(C2=CC=C(C=C2)C(=O)NCCC(=O)O)NC3=CN(N=C3)C4=CC=C(C=C4)C(F)(F)F
	CHEMBL1933360	CCCC(C1=CC=C(C=C1)C(=O)NCCC(=O)O)OC2=CN(N=C2)C3=CC=C(C=C3)C(F)(F)F
	CHEMBL198736	CC(C)(C)C1CCC(CC1)N(CC2=CC=C(C=C2)C(=O)NCCC(=O)O)C(=O)NC3=CC=C(C=C3)OC(F)(F)F

[1] Values were extracted from the Pubchem database.

[2] Here, the IC50 value was deposited in the Pubchem database.

Table B. Clustal Omega identity scores computed between target and template sequences used in this study.

Target – Uniprot entry	Template – Uniprot entry	Sequence identity score [%]
GLR_HUMAN - P47871	GLP1R_HUMAN - P43220	47.23
	GIPR_HUMAN - P48546	50.44
	VIPR1_HUMAN - P32241	36.22
	PACR_HUMAN - P41586	35.68

GLP1R_HUMAN - P43220	GIPR_HUMAN - P48546	45.82
	VIPR1_HUMAN - P32241	39.12
	PACR_HUMAN - P41586	33.89
GIPR_HUMAN - P48546	VIPR1_HUMAN - P32241	37.13
	PACR_HUMAN - P41586	34.43
VIPR1_HUMAN - P32241	PACR_HUMAN - P41586	49.54

Figure A. GUT-DOCK results for compound 15 – Autodock VINA-generated binding affinities for GLP1R. Here, the binding affinity of compound 15 was compared with precomputed binding affinities of other beta-blockers (see Table 3 in the main text of the manuscript) for GLP1R. Compound 15 was docked to the allosteric binding site of GLP1R.

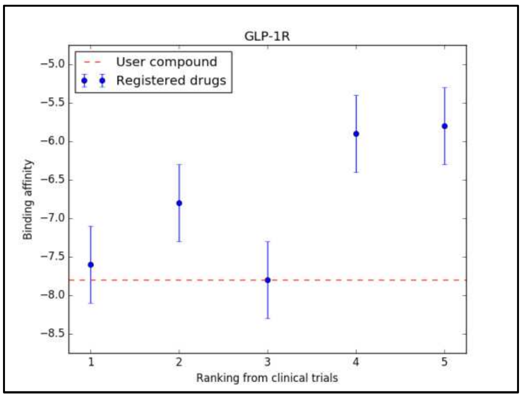

Figure B. GUT-DOCK results for compound 15 – a Ligplot-generated visualization of interactions with GLP1R. Here, standard Ligplot settings for hydrogen bonds were used. Compound 15 was docked to the allosteric binding site of GLP1R.

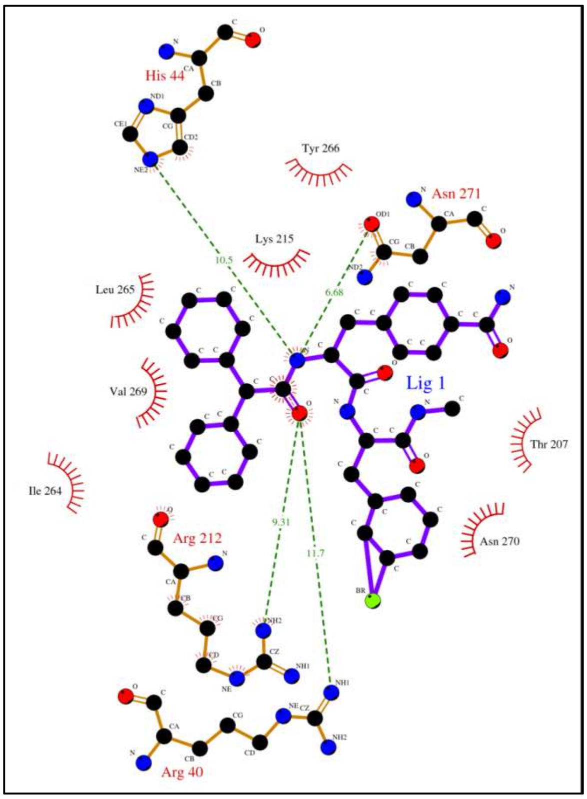

Figure C. The binding mode of compound 15 to GLP1R obtained with GUT-DOCK. Here, GLP1R was used as a GPCR receptor, left – extracellular view of the orthosteric site, right – side view of the allosteric site. Class B GPCR conserved residues were depicted. Compound 15 located in the orthosteric site (the Autodock VINA score equal to -10.5) formed hydrogen bonds (yellow dashed lines) with Q3.37 and R5.40. Compound 15 located in the allosteric site (the Autodock VINA score equal to -7.8) formed hydrogen bonds with R6.37, N8.51and R2.55 and polar contact with S6.41.

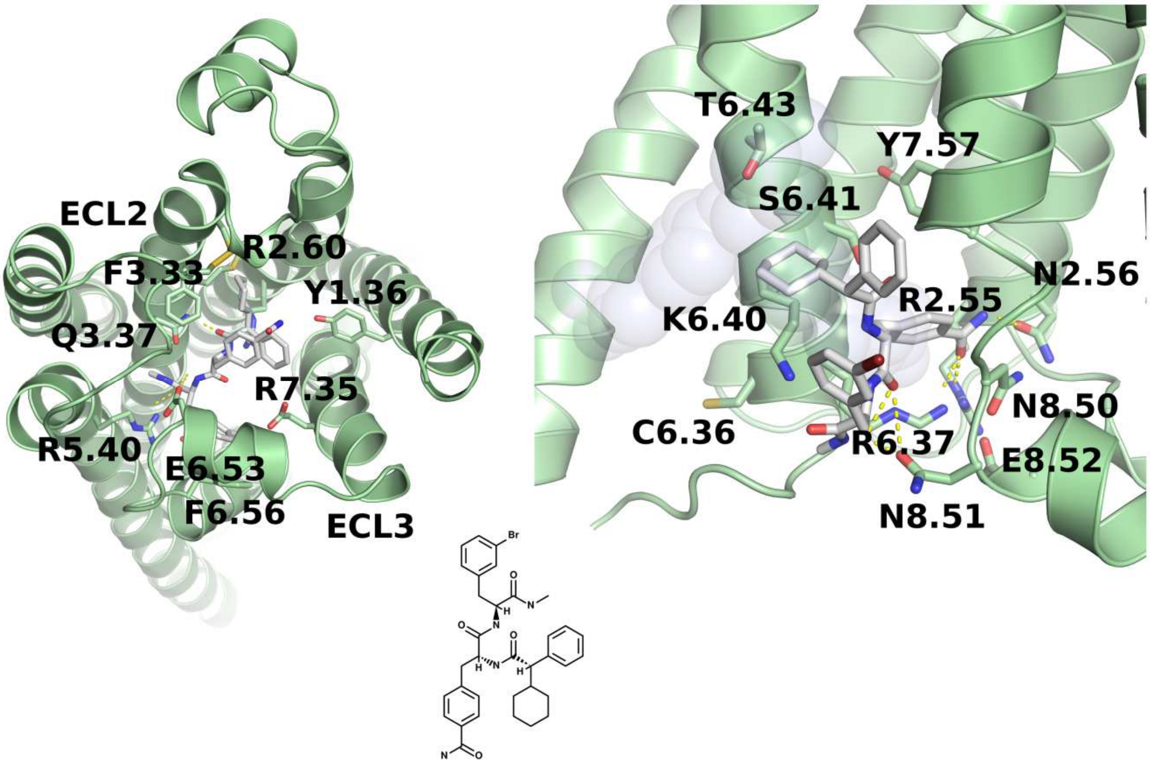

Supplement: S1 File — A file including: active compounds lists, sequence identity between templates and targets from the current study, figures presenting GUT-DOCK results for compound 15 and GLP1R. (PDF) [file pone.0210705.s001.pdf]
